# Non-invasive Diagnostic Tests in Cystic Fibrosis-Related Liver Disease: A Diagnostic Test Accuracy Network Meta-Analysis

**DOI:** 10.3389/fmed.2021.598382

**Published:** 2021-07-27

**Authors:** Ágnes Rita Martonosi, Alexandra Soós, Zoltán Rumbus, Péter Hegyi, Vera Izsák, Piroska Pázmány, Marcell Imrei, Szilárd Váncsa, Zsolt Szakács, Andrea Párniczky

**Affiliations:** ^1^Institute for Translational Medicine, Medical School, University of Pécs, Pécs, Hungary; ^2^Heim Pál National Paediatric Institute, Budapest, Hungary; ^3^Doctoral School of Clinical Medicine, University of Szeged, Szeged, Hungary; ^4^János Szentágothai Research Centre, University of Pécs, Pécs, Hungary

**Keywords:** cystic fibrosis, transient elastography, TE, cystic fibrosis-related liver disease, network meta-analysis, non-invasive diagnostic methods

## Abstract

**Background and Aims:** Cystic fibrosis-related liver disease (CFLD) is one of the leading causes of morbidity and mortality in cystic fibrosis (CF). Several non-invasive diagnostic methods have been proposed as screening tools for CFLD. Our aim was to rank all available non-invasive modalities for diagnostic performance.

**Methods:** A systematic search was performed in five medical databases to find studies which reported on any single or composite non-invasive diagnostic test (as an index test) compared to the Debray, the EuroCare or the Colombo criteria (as a reference standard). Ranking was carried out with a Bayesian diagnostic test accuracy network meta-analysis based on superiority indices, calculated for pooled sensitivity (Se) and specificity (Sp) with a 95% confidence interval (CI). The study was registered under CRD42020155846 in PROSPERO.

**Results:** Fifteen studies with 15 index tests and a combination of them were included. The New criteria proposed by Koh et al. – which represent a composite diagnostic definition for CFLD including liver biochemistry, ultrasonography, transient elastography and fibrosis markers—had the best performance for detecting CFLD (Se:94%[CI:58–100], Sp:72%[CI:52–84]); while transient elastography (Se:65%[CI:56–74], Sp:88%[CI:84–91]) and a combination of it with a tissue inhibitor of metalloproteinase-4 measurement (Se:78%[CI:30–100], Sp:64%[CI:18–95%]) proved to be the second and third best options, respectively. In the imaging techniques subgroup, transient elastography (Se:66%[CI:57–72], Sp:88%[CI:85–91%]), acoustic radiation force impulse in the right lobe (Se:54%[CI:33–74], Sp:88%[CI:66–96]) and that in the left lobe (Se:55%[CI:23–81], Sp:82%[CI:50–95]) were ranked the highest. Comparing biochemical markers/fibrosis indices, the measurement of the Forns index (Se:72%[CI:25–99], Sp:63%[CI:16–94]), the aspartate aminotransferase-to-platelet ratio (Se:55%[CI:41–68], Sp:83%[CI:66–89]) and alkaline phosphatase (Se:63%[CI:18–93], Sp:64%[CI:19–95]) were ranked the highest.

**Conclusion:** The New criteria show the best diagnostic performance. In clinical practice, transient elastography seems to be a simple, cheap and non-invasive tool, outperforming imaging, biochemical and fibrosis tests for detecting CFLD. Further studies are needed to validate our findings.

## Introduction

Cystic fibrosis (CF) is an autosomal recessive genetic disorder, caused by mutations in the gene which encodes the cystic fibrosis transmembrane conductance regulator protein (CFTR). CFTR is a cyclic adenosine monophosphate (cAMP)-regulated epithelial cell membrane ion channel. Dysfunction of chloride transport results in thick, viscous mucus production mainly in the lungs, sweat glands, digestive system, and reproductive organs (1, 2).

With the great improvement in medical care and the treatment of pulmonary complications leading to ever-increasing life quality and expectancy, there is a rising number of CF patients with gastrointestinal involvement which has a major impact on morbidity and mortality (3–5). In addition to pancreatic insufficiency, hepatic relation, which is recognized as one of the leading non-pulmonary causes of death in CF, has shown an upward tendency with a current prevalence of 40% (6). The non-specifically used term—cystic fibrosis-related liver disease (CFLD)—covers a multiplicity of hepatobiliary disorders, including elevated liver biochemical markers, cholestasis, biliary tract malformations (e.g., sclerosing cholangitis, cholelithiasis, micro-gallbladder, and gallbladder dyskinesia), steatosis, fibrosis and cirrhosis, leading to portal hypertension, and its complications (4, 7–9).

Due to the subclinical nature and the heterogeneity of liver diseases, early regular screening for CFLD is required to identify not only portal hypertension-related life-threatening liver complications, but also asymptomatic hepatic impairments (10).

As a result of the clinical diversity of CFLD, the diagnostic definition is still open to discussion. Liver biopsy can be regarded as the gold standard invasive diagnostic method for CFLD, although there are several disadvantages to it, including significant morbidity, mortality, costs and modest diagnostic performance for patchy liver involvement.

“The Debray” CFLD criteria currently represent the most acknowledged non-invasive diagnostic tool (10–15); however, they are composed of several different diagnostic modalities. They consist of physical examination, liver biochemistry measurements, ultrasonography, and liver biopsy in the event of diagnostic doubt. “The Colombo” or “the Eurocare” criteria are mainly based on the same diagnostic algorithms, but they do not include liver biopsy. In 2017 Koh et al. developed a new diagnostic criteria for CFLD involving transient elastography and non-invasive fibrosis markers for the diagnostic process but excluding physical examination (for further information about the definitions, see Supplementary Table 3).

Although, there has been a great improvement in CFLD diagnostics, an accurate, cost-effective, easy-to-use, non-invasive screening technique is still needed. Since the relative diagnostic performance of all available non-invasive screening modalities has not been determined in its full complexity, our aim was to evaluate and rank tests as well as diagnostic strategies for diagnosing CFLD in a diagnostic test accuracy network meta-analysis (DTA-NMA). DTA-NMA is a novel meta-analysis technique which allows multiple diagnostic tests to be interpreted in a single analysis and a comparison of numerous screening techniques in the absence of head-to-head comparisons (16). Findings from DTA-NMAs might guide clinicians and societies in the field of science dealing with CF to improve diagnostics in CFLD.

## Methods

The study is reported as per the Preferred Reporting Items for Systematic Review and Meta-Analysis of Diagnostic Test Accuracy Studies (PRISMA-DTA) Statement (17) and Network Meta-Analyses (PRISMA-NMA) (18) (*for details, see* Supplementary Table 1).

### Search, Selection, and Eligibility

A systematic literature search was conducted by two independent review authors (ÁRM and VI) in MEDLINE (via PubMed), EMBASE, the Web of Science Core Collection, CENTRAL, and Scopus using the combination of the following keywords “cystic fibrosis” and “liver” *(for details, see* Supplementary Table 2*)* on 8 October 2019. Title and abstract field filters were applied in Scopus and Web of Science. Otherwise, no additional filters or restrictions were used based on language, publication year, or country of origin. A manual search was also performed for articles cited in the included studies to locate publications absent from the original search strategy.

Records were managed with EndNote X9.1.1 software (2020 Clarivate™ Analytics, Philadelphia, PA, USA). A standard three-step selection by title, abstract and full text was applied, with any disagreements resolved by a third independent senior review author (ZS).

Eligible study populations consisted of adult and pediatric CF patients. We compared the diagnostic performance of different non-invasive diagnostic modalities for CFLD—as index tests—to the Debray criteria or similar ones (the EuroCare or the Colombo criteria), as the reference standard (for definitions, see Supplementary Table 3) (6, 10–12).

The non-invasive diagnostic methods were divided into two subgroups, including imaging techniques (transient elastography (TE) [Fibroscan® (Echosens, Paris)], acoustic radiation force impulse (AFRI) in the left and right lobes, and two-dimensional shear wave elastography (2D-SWE)) and biochemical tests/fibrosis indices [hepascore, Forns index, fibrotest, fibrotest corrected by haptoglobin, aspartate aminotransferase-to-platelet ratio index (APRI), fibrosis-4 index (FIB-4), and aspartate aminotransferase-to-alanine aminotransferase ratio (AAR)] and biochemical markers [aspartate aminotransferase (AST), alanine aminotransferase (ALT), γ-glutamyl-transferase (GGT), and alkaline phosphatase (ALP)] (for further information about the index tests, see Supplementary Table 4). Eligible studies had to provide data on at least one index test or a combination of index tests. We also included the New criteria proposed by Koh et al. as a composite diagnostic modality (19).

In the case of potentially overlapping study populations (based on authors, sites and index tests), those with a larger study population were included.

We incorporated cohort or case-control studies published as full-text papers or conference abstracts to reduce selection bias.

### Data Extraction and Statistical Analysis

The data were extracted by two independent review authors (ÁRM and VI) into a purpose-designed data collection table, with any disagreement resolved through third-party arbitration by a senior review author (ZS). Then, 2 × 2 contingency tables were constructed with raw data for true positive, true negative, false positive and false negative values. If data for more than one cut-off value were reported for the same index test within a study, we chose those calculated with the best cut-off value according to Youden's index.

We performed a Bayesian DTA-NMA to investigate which non-invasive diagnostic method may be the best choice for diagnosing CFLD. This method allows us to make direct and indirect pairwise comparisons of relative performance when a common comparator—a reference standard—is given. We contemplated the use of diagnostic odds ratios (DORs); however, results proved to be uninterpretable due to continuity correction. Finally, we decided to rank index tests according to superiority indices (SIs) (20). SIs vary between 0 and ∞; the larger the SI, the more accurately a screening test is expected to predict the target condition compared to other screening tests, based on relatively better simultaneous performance of both assessment measures. If the SIs tend toward 1, it means that the index tests are equal (21).

All the statistical calculations were performed by using the ANOVA arm-based model (21) and R programming language (R Core Team, 2019, Vienna, Austria, R version 3.6.1) (22) with the rstan, loo and plyr packages (22–24). We illustrated the network graph using STATA (version 15.1).

To display the networks, we created and designed graphs where nodes are associated with different non-invasive diagnostic techniques and edges (represented by solid black lines) serve as head-to-head (direct) comparisons. The size of the nodes is proportional to the number of studies evaluating each diagnostic test, and the thickness of the lines between the nodes is proportional to the number of each direct comparison. We created four networks evaluating all the diagnostic tests; combined tests and the New criteria; imaging modalities; and biochemical markers and fibrosis indices.

### Risk of Bias and Applicability Assessment

The risk of bias and applicability of the diagnostic studies were evaluated by two independent review authors (ÁRM and PP) using the Quality Assessment of Diagnostic Accuracy Studies-2 (QUADAS-2). The result of the assessment was graphically demonstrated by Review Manager (RevMan Web, version: 5.3. The Cochrane Collaboration, 2014). Concerns about risk of bias and applicability were rated as “low,” “high,” or “unclear.” The unclear category was used when incomplete data were reported. Any disagreements were resolved by consensus among the review authors (25).

### Pre-study Protocol

The DTA-NMA was based on a protocol previously registered in PROSPERO on 4 January 2020 under registration number CRD42020155846. Liver biopsy was originally planned as the reference standard. After completing the literature search, we realized that we had limited data on liver biopsy (liver biopsy was performed only in 2% of the patients examined—mainly in the case of diagnostic doubt), so we deviated from our protocol and chose the Debray, EuroCare, and Colombo criteria as the reference standard. Further, we performed a *post-hoc* subgroup analysis by type of test (that is, imaging techniques, fibrosis indices and biochemical markers).

## Results

### Study Selection

**Figure 1:**
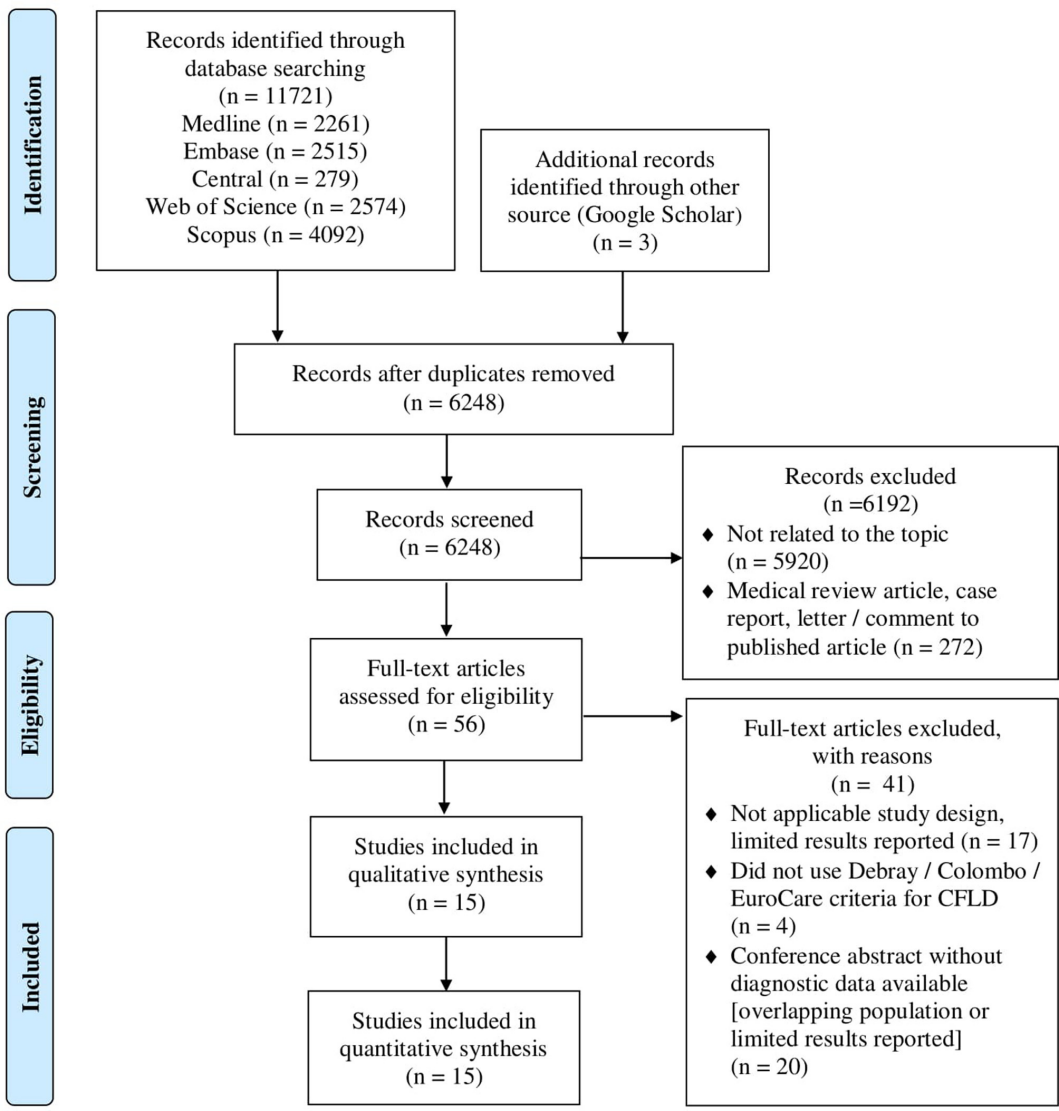
PRISMA 2009 flow diagram of the study selection. The algorithm for the study selection; out of 11,721 records, 13 full-text articles and two conference abstracts were included in the final analysis.

Out of 11,721 records, a total of 53 articles were assessed for eligibility by full text, of which 15 studies (13 full-text papers and two conference abstracts) were used in the meta-analysis. The flowchart for the process and reasons for exclusions on full-text assessment are shown in Figure 1.

### Study Characteristics

The characteristics of the included studies are reported in Table 1. Studies took place in Greece, Spain, Italy, Germany, the UK, Belgium, the USA, Canada, and Australia. Recruitment was arranged between 1980 and 2018. Thirteen studies were prospective (11, 12, 19, 26–30, 32–36), one was a retrospective cohort study (37), and one was a case control study (31). Four studies examined pediatric patients (11, 27, 28, 32), another six only assessed adults (19, 26, 30, 31, 35, 36), and the remaining five had a mixed-aged population (12, 29, 33, 34, 37). A total of 1 403 patients—constituting 383 CFLD patients (27.2%)—were involved in the network meta-analysis. The studies included 15 different index tests or a combination of them, of which TE was the most widely used (examined in 10 studies). A summary of the baseline study characteristics with the raw data collected is presented in Table 2.

**Table 1:** Baseline characteristics of the included studies.

**First Author**	**Country (centers)**	**Recruitment period**	**Study design**	**Population**	**Number of CF patients (Percentage of CFLD %)**	**Reference standard**
Alexopoulou et al. (26)	Greece (single)	2014–2017	Prospective cross-sectional	Adult	62 (25.8)	Debray
Calvopina et al. (27)	Australia (single)	2015–2018	Prospective cross-sectional	Pediatric	97 (56.7)	EuroCare
Canas et al. (28)	Spain (single)	2015	Prospective cross-sectional	Pediatric	72 (31.9)	Colombo
Colombo et al. (12)	Italy (single)	1980–1990	Prospective cross-sectional	Adult and pediatric	177 (27.1)	Colombo
Friedrich-Rust et al. (29)	Germany (single)	2009–2012	Prospective cross-sectional	Adult and pediatric	106 (22.6)	Debray
Karlas et al. (30)	Germany (single)	2010	Prospective cross-sectional	Adult	55 (25.4)	Colombo
Kitson et al. (31)	Australia (single)	2009–2010	Case-control	Adult	50 (50)	Colombo
Koh et al. (19)	USA (single)	No data	Prospective longitudinal	Adult	36 (22.2)	Debray
Lam et al. ^*^(11)	Canada (single)	No data	Prospective cross-sectional	Pediatric	41 (9.7)	EuroCare
Lewindon et al. (32)	Australia (single)	2011–2016	Prospective cross-sectional	Pediatric	138 (23.9)	Debray
Rath et al. ^#^(33)	Germany (single)	2008–2010	Prospective cross-sectional	Adult and pediatric	145 (46.8)	Colombo
Rath et al. ^#^(34)	Germany (single)	2008–2010	Prospective cross-sectional	Adult and pediatric	45 (37.7)	Colombo
Sadler et al. (35)	Canada (single)	2010–2011	Prospective cross-sectional	Adult	127 (14.1)	Debray
Scott et al. ^*^(36)	UK (single)	No data	Prospective cross-sectional	Adult	102 (9.8)	Debray
Van Biervliet et al. (37)	Belgium (single)	2007–2013	Retrospective	Adult and pediatric	150 (13.3)	Debray

*13 full-text articles and two conference abstracts were included in the analysis with a total of 1 403 cystic fibrosis patients—including 383 CFLD patients—from nine countries*.

* *Conference abstract*,

# *Potentially overlapping population*.

*CF, cystic fibrosis; CFLD, cystic fibrosis-related liver disease*.

**Table 2:** Summary of the diagnostic modalities with index tests and raw data.

**First Author**	**Index tests (Unit)**	**TP**	**FP**	**FN**	**TN**
Alexopoulou et al. (26)	APRI−0.43 (–)	5	0	11	46
	TE−6.8 (kPa)	8	6	8	40
	AAR−1.00 (–)	7	17	9	29
	New criteria^*^	16	11	0	35
Calvopina et al. (27)	2D-SWE−6.81(kPa)	41	12	14	29
	2D-SWE−6.81 (kPa) + APRI (no data)	24	2	12	17
Canas et al. (28)	ARFI right lobe−1.27 (m/s)	13	5	10	44
	US^†^	11	10	12	39
Colombo et al. (12)	Liver function tests: ALT, AST, and GGT (IU/l)^‡^	21	8	26	66
Friedrich-Rust et al. (29)	TE−7.10 (kPa)	6	6	8	61
	ARFI right lobe−1.42 (m/s)	13	5	11	77
	ARFI left lobe−1.45 (m/s)	13	11	11	70
	FIBROTEST−0.21 (–)	9	8	15	74
	FIBROTEST corrected by haptoglobin−0.27 (–)	9	10	15	72
	US^§^	23	43	1	39
Karlas et al. (30)	APRI−0.23 (–)	12	12	2	29
	TE−5.9 (kPa)	6	1	8	34
	ARFI right lobe−1.28 (m/s)	6	3	8	37
	ARFI left lobe−1.43 (m/s)	7	4	7	36
	FORNS−2.15 (–)	13	16	1	25
Kitson et al. (31)	TE−6.80 (kPa)	19	2	6	23
Koh et al. (19)	New criteria^*^	8	9	0	19
Lam et al. (11)	TE−5.3 (kPa)	4	5	0	32
Lewindon et al. (32)	APRI−0.27 (–)	16	34	7	38
	TE−5.55 (kPa) +APRI −0.27 (–)	20	19	3	53
	TE−5.55 (kPa)	23	19	10	86
Rath et al. (33)	US^||^	48	25	20	52
	TE−5.5 (kPa)	36	14	32	63
Rath et al. (34)	TE−6.3 (kPa)	14	0	3	28
	TIMP-4−1,603 (pg/ml)	11	5	6	23
	ENG−8.6 (ng/ml)	12	8	5	20
	APRI 0.133 (–)	8	1	9	27
	ALP^¶^	12	5	5	23
	TIMP-4−1,603 (pg/ml) +Endoglin−8.6 (ng/ml)	15	13	2	15
	TE +ENG−6.3+8.6	15	8	2	20
	TE−6.3 (kPa) +TIMP-4 −1,603 (pg/ml)	17	5	0	23
Sadler et al. (35)	APRI−0.5 (–)	9	6	9	98
	TE−5.3 (kPa)	12	19	6	90
	FIBROTEST−0.1 (–)	14	38	3	51
Scott et al. (36)	New criteria^*^	10	23	0	69
Van Biervliet et al. (37)	TE−6.81 (kPa)	18	11	2	119

* *Definition of the New criteria by Koh et al. (19)*,

† *Ultrasound severity score (28)*,

‡ *Liver function tests: elevation above the upper normal limits of the levels of at least two serum liver enzymes (AST, ALT, and GGT) confirmed after 6 months (12)*,

§ *Ultrasound severity score (29)*,

|| *Ultrasound severity score (11)*,

¶ *ALP: age- and gender-specific cut-off of alkaline phosphatase, with values determined by the Department for Laboratory Medicine and Clinical Chemistry of the University Hospital Giessen according to the International Federation of Clinical Chemistry. TP, true positive value; FP, false positive value; FN, false negative value; TN, true negative value; TE, transient elastography; 2D-SWE, two dimensional shear wave elastography; ARFI, acoustic radiation force impulse; US, ultrasonography; ALP, alkaline phosphatase; AST, aspartate aminotransferase; ALT, alanine aminotransferase; GGT, γ- glutamyl transferase; APRI, AST-to-platelet-ratio index; AAR, AST-to-ALT ratio; TIMP-4, metalloproteinase inhibitor-4; ENG, endoglin*.

### Synthesis

**Figure 2:**
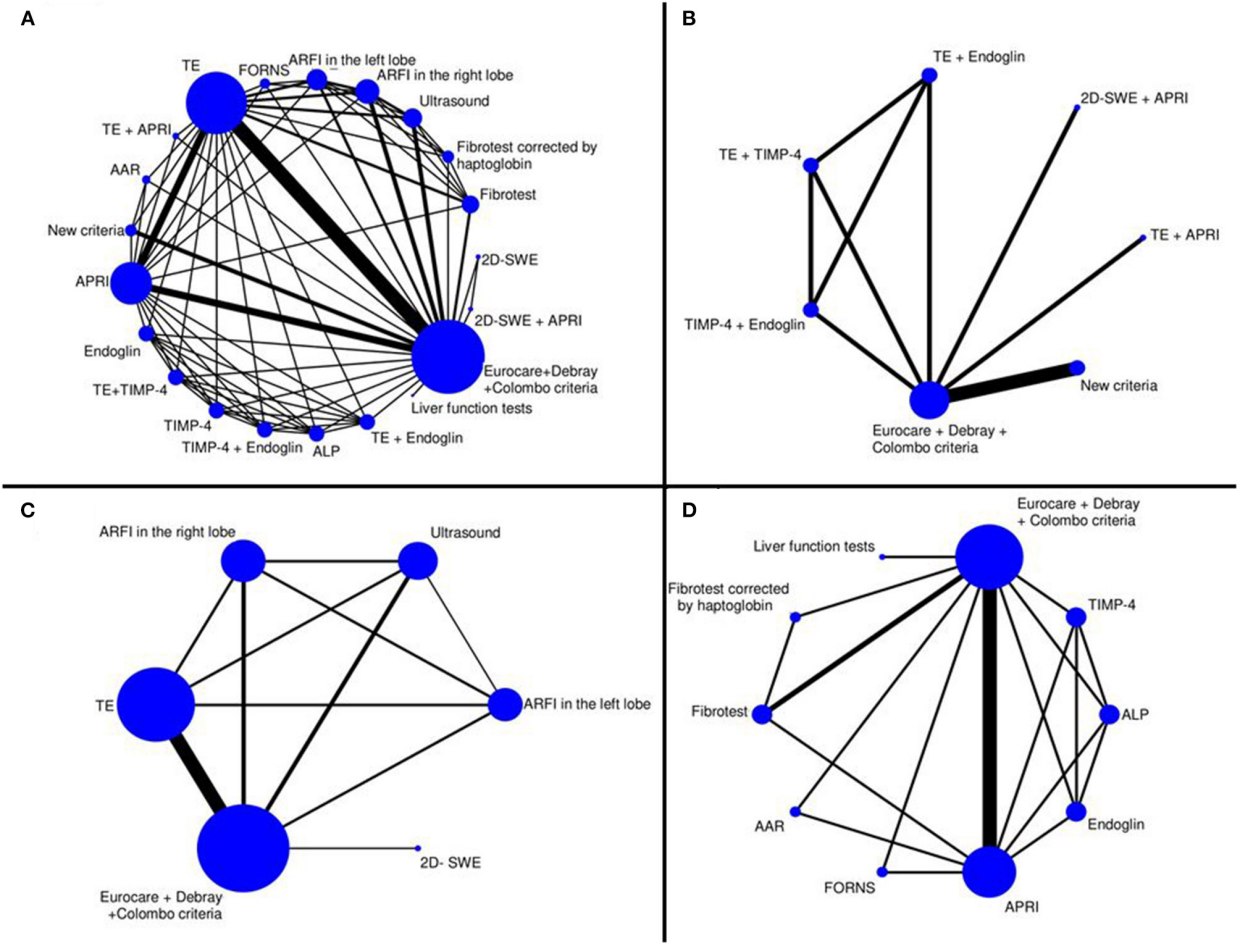
Network graphs for the different diagnostic modalities. To display the created network we created, we designed graphs where nodes are associated with different non-invasive diagnostic techniques and edges (represented by solid black lines) serve as head-to-head (direct) comparisons. The size of the nodes is proportional to the number of studies evaluating each diagnostic test, and the thickness of the lines between the nodes is proportional to the number of each direct comparison. **(**Network **A)** Comparison of all diagnostic tests (*n* = 20) to the reference standard. **(**Network **B)** Comparison of the combined tests and the New criteria to the reference standard (*n* = 6). **(**Network **C)** Comparison of tests based on imaging only (*n* = 5). **(**Network **D)** Comparison of biochemical markers and fibrosis indices (*n* = 9) to the reference standard. TE, transient elastography; APRI, AST-to-platelet-ratio index; AAR, AST-to-ALT ratio; TIMP-4, tissue inhibitor of Metalloproteinase-4; ALP, alkaline phosphatase; Liver function tests; AST, aspartate aminotransferase; ALT, alanine aminotransferase; GGT, γ-glutamyl-transferase; 2D-SWE, two-dimensional shear wave elastography; ARFI, acoustic radiation force impulse.

The top three diagnostic modalities are shown in Table 3, ranking for all the tests is presented in Supplementary Table 5, and network graphs (Networks A–D) are displayed in Figure 2. Different analyses were performed in the four networks. Network A represents a comparison of all the index tests to the reference standard. Network B entails a comparison between the combined index tests, the New criteria and the reference standard. Network C compares imaging techniques, and Network D examines biochemical markers and fibrosis indices in comparison with the reference standard.

**Table 3:** Ranking of the top three index tests by superiority indices.

**Raking of the index tests**	**Index tests**	**SI (95% CI)**	**Pooled Se % (95% CI)**	**Pooled Sp % (95% CI)**
**Network A**				
#1	The New criteria	16.22 (0.64–31)	94 (58–100)	72 (52–84)
#2	TE	10.66 (1.40–27)	65 (56–74)	88 (84–91)
#3	TE + TIMP-4	8.84 (0.03–35)	78 (30–100)	64 (18–95)
**Network B**				
#1	The New criteria	4.29 (0.14–11)	80 (54–94)	78 (50–93)
#2	TE + TIMP-4	3.97 (0.11–11)	81 (33–100)	66 (19–96)
#3	2D-SWE + APRI	2.01 (0.09–9)	61 (18–94)	70 (23–99)
**Network C**				
#1	TE	3.85 (0.33–9)	66 (57–72)	88 (85–91)
#2	ARFI right lobe	2.33 (0.2–7)	54 (33–74)	88 (66–96)
#3	ARFI left lobe	1.60 (0.14–7)	55 (23–81)	82 (50–95)
**Network D**				
#1	FORNS	5.23 (0.08–17)	72 (25–99)	63 (16–94)
#2	APRI	4.36 (0.20–13)	55 (41–68)	83 (66–89)
#3	ALP	3.89 (0.08–15)	63 (18–93)	64 (19–95)

***Network A:** The first analysis includes all the eligible studies (fifteen) with all the different index tests (fifteen) and combinations of them. The New criteria represent the relatively best diagnostic modality for detecting CFLD, while TE and a combination of TE + TIMP-4 proved to be the second and third best options, respectively. **Network B:** In the second network analysis, in which the New criteria and the combined index tests were evaluated, the New criteria took first place, while a combination of TE + TIMP-4 and 2D-SWE + APRI ranked second and third, respectively. **Network C:** Network C shows the comparison of imaging-based techniques. TE was ranked the highest, followed by ARFI in the right and left lobes. **Network D:** This network represents the ranking of biochemical markers and fibrosis indices, which revealed that the Forns index seems to be the relatively best diagnostic method, while APRI and ALP took second and third place, respectively. More detailed ranking can be found in Supplementary Table 4. SI, superiority index; Se, sensitivity; Sp, specificity; TE, transient elastography; 2D-SWE, two-dimensional shear wave elastography; APRI, aspartate aminotransferase-to-platelet ratio index; ARFI, acoustic radiation force impulse; ALP, alkaline phosphatase*.

Network A (Figure 2, Graph **A**) summarizes the results of the 15 included studies with all the index tests. Ranked by superiority indices, the New criteria (mean SI: 16.22; 95% CI: 0.64–31) represent the relatively best diagnostic method for detecting CFLD with a sensitivity of 94% (95% CI: 58–100%) and a specificity of 72% (95% CI: 52–84%), while TE (mean SI: 10.66; 95% CI: 1.40–27) proves to be the second best option with a sensitivity of 65% (95% CI: 56–74%) and a specificity of 88% (95% CI: 84–91%). A combination of TE and TIMP-4 (mean SI: 8.84; 95% CI: 0.03–35) takes third place in diagnosing CFLD with a sensitivity of 78% (95% CI: 30–100%) and a specificity of 64% (95% CI: 18–95%). A combination of TE and TIMP-4 provides higher pooled sensitivity but lower pooled specificity than TE alone.

If we compare the combined index tests and the New criteria to the reference standard in the second network (Figure 2, Graph **B**), the New criteria (mean SI: 4.29; 95% CI: 0.14–11) form the relatively best choice for detecting CFLD with a sensitivity of 80% (95% CI: 54–94%) and a specificity of 78% (95% CI: 50–93%), while a combination of TE and TIMP-4 (mean SI: 3.97; 95% CI: 0.11–11), with a sensitivity of 81% (95% CI: 33–100%) and a specificity of 66% (95% CI: 19–96%), and a combination of 2D-SWE and APRI (mean SI: 2.01; 95% CI: 0.09–9), with a sensitivity of 61% (95% CI: 18–94%) and a specificity of 70% (95% CI: 23–99%), are ranked the second and third best options, respectively.

Network C (Figure 2, Graph **C***)* represents a comparison of imaging techniques to the reference standard. TE (mean SI: 3.85; 95% CI: 0.33–9) is ranked the highest with a sensitivity of 66% (95% CI: 57–72%) and a specificity of 88% (95% CI: 85–91%), while ARFI in the right lobe (mean SI: 2.33; 95% CI: 0.27–7), with a sensitivity of 54% (95% CI: 33–74%) and a specificity of 88% (95% CI: 66–96%), and ARFI in the left lobe (mean SI: 1.60; 95% CI: 0.14–7), with a sensitivity of 55% (95% CI: 23–81%) and a specificity of 82% (95% CI: 50–95%), prove to be the second and third best options, respectively.

If we rank biochemical markers and fibrosis indices by SIs (Figure 2, Graph **D**), the Forns index (mean SI: 5.23; 95% CI: 0.08–17) is ranked the best with a sensitivity of 72% (95% CI: 25–99%) and a specificity of 63% (95% CI: 16–94%), while APRI (mean SI: 4.36; 95% CI: 0.20–13), with a sensitivity of 72% (95% CI: 25–99%) and a specificity of 83% (95% CI: 66–89%), and ALP (mean SI: 3.89; 95% CI: 0.08–15), with a sensitivity of 63% (95% CI: 18–93%) and a specificity of 64% (95% CI: 19–95%), take second and third place, respectively. However, the Forns index is ranked the best by SI; it has a higher pooled sensitivity but a lower pooled specificity than APRI or ALP.

### Risk of Bias and Applicability Assessment

**Figure 3:**
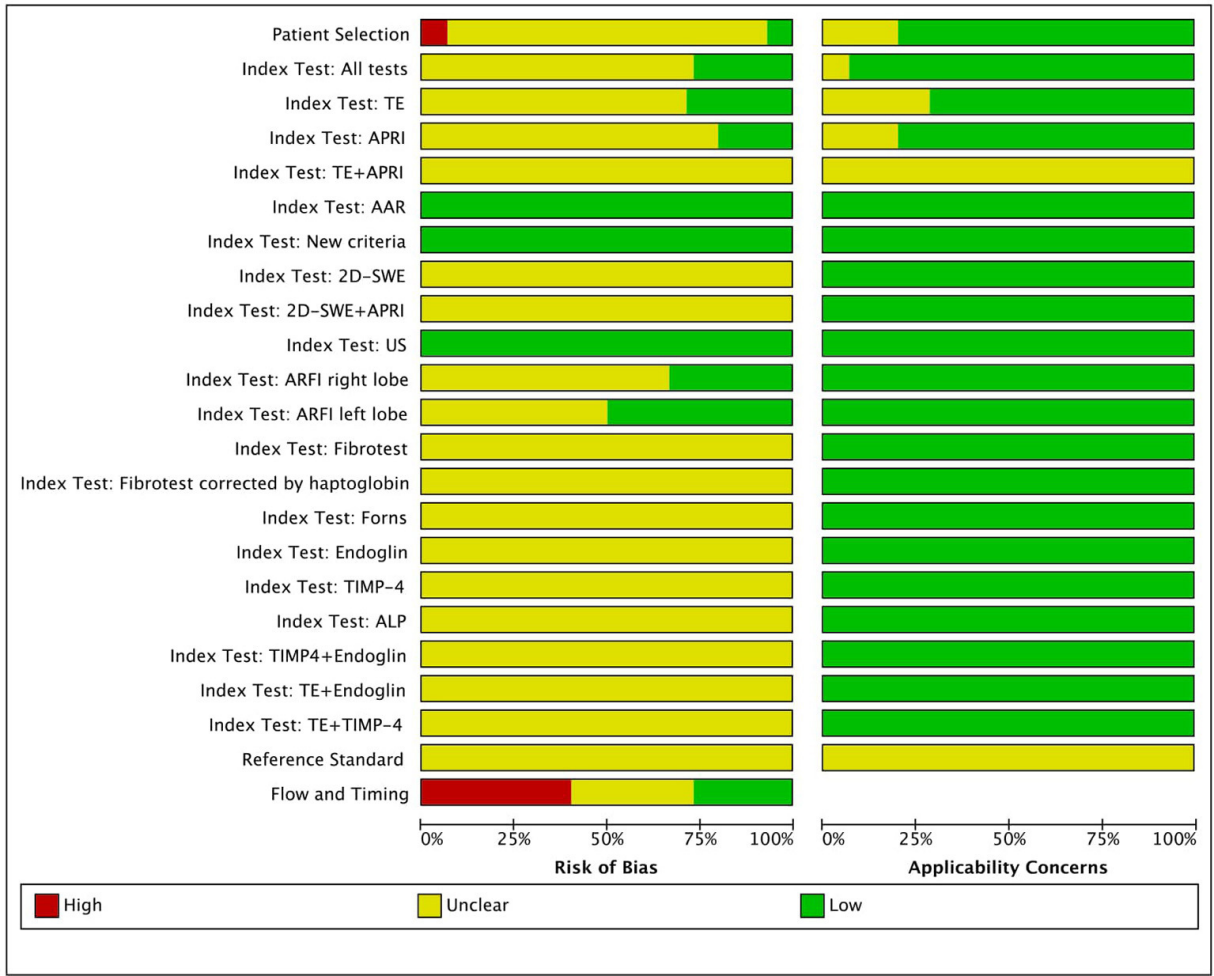
Risk of bias and applicability assessment. TE, transient elastography; APRI, AST-to-platelet-ratio index; AAR, AST-to-ALT ratio; 2D-SWE, two-dimensional shear wave elastography; US, ultrasonography; ARFI, acoustic radiation force impulse; TIMP-4, tissue inhibitor of metalloproteinase-4; ALP, alkaline phosphatase.

A summary of the risk of bias and applicability assessment is presented in Figure 3. The majority of the studies (*n* = 13) had an unclear risk of bias as regards patient selection due to poor reporting of the selection process [the single case-control study was rated as having a high risk of bias (31)]. The cut-off values for the index tests were not pre-specified (*n* = 9) and were interpreted with the knowledge of the reference standard in the studies. The latter problem was observed as regards the reference standard in 11 studies, while we must admit minor differences in the sets of criteria used as a reference standard (although the Debray, the EuroCare, and the Colombo criteria are based on the same diagnostic algorithm; for definitions, see Supplementary Table 3). In 40% of the studies (*n* = 6) flow and timing field were deemed high risk due to the unknown time interval between the index tests and reference standard or the discrepancy between the sample size of the recruited population and that of the population analyzed. All the studies had low to unclear applicability concerns (for more details, see Supplementary Figure 1).

## Discussion

### Summary of Evidence

Our DTA-NMA ranks the currently available non-invasive screening methods for diagnosing CFLD by diagnostic performance.

Recent guidelines promote annual screening for hepatic involvement in the CF population. Despite the questionable objectivity, the current recommendation is to perform a routine physical examination, liver biochemical tests, and ultrasonography (10, 38). Our results will be discussed in the context of a Best practice guideline (10, 39).

### The Best Practice Guideline and New Diagnostic Methods

The first diagnostic criterion is physical examination. Finding hepatomegaly during a clinical evaluation is non-invasive, inexpensive and accessible but can result in interobserver variability.

The second step of the diagnostic work-up is the assessment of liver function tests. Routine blood tests are relatively inexpensive, but anxiety-provoking and time-consuming. Furthermore, they can be inconvenient due to the blood-taking procedure and non-specific with low sensitivity and specificity. Occasional fluctuations in liver transaminase levels can be observed during infection or administration of medication, even in malnutrition as well (40); therefore, they might not correlate with the severity of the disease (9). GGT might achieve better than AST or ALT in identifying liver nodularity, but the range of the biochemical thresholds is still under discussion (41).

The ultrasound scan is the third part of the diagnosis. Abdominal ultrasound is regarded as a valuable marker in detecting CFLD, since it is non-invasive, simple to perform when used by an expert radiologist, inexpensive, and more sensitive than a physical examination or biochemical tests (42, 43), although it also can produce intra- and interobserver variability. Moreover, normal ultrasound does not rule out the presence of hepatic involvement (especially fibrosis) (44).

In the case of diagnostic uncertainty, liver biopsy can be confirmatory in the protocol. Histological assessment is regarded as the gold standard invasive diagnostic method. The main disadvantages are that it is invasive, relatively costly and inconvenient; furthermore, it is associated with anxiety, bleeding, significant morbidity, and mortality. Moreover, due to the patchy liver involvement, it may underestimate the severity of the lesions (it might elevate the risk for false negative results), and there is also has a lack of information about the speed of disease progression (45).

The continuous improvement of non-invasive methodologies gradually limits the indications of liver biopsy. Transient elastography is a quantitative method based on a one-dimensional image of liver tissue stiffness with a combination of the use of ultrasound and low-frequency elastic wave. The role of TE in detecting early changes in the liver tissue is under evaluation. However, a large number of good-quality studies report fine diagnostic accuracy for advanced fibrosis and cirrhosis (11). In addition, TE is a cost-effective intervention (46). Elevated body mass index, obesity or ascites can disturb the accuracy of the measurements. Oedema, inflammation, deep breath, the Valsalva maneuver or meal intake can influence liver stiffness, so it should be performed after fasting, while the patient is holding his/her breath. The nature of hepatic involvement, age, gender etc. might affect cut-off thresholds as well (47).

Controlled attenuation parameter, which is based on the attribution of ultrasonic signals by TE, can raise the accuracy of steatosis detection. This method is relatively fast, reliable and reproducible and has good intra- and interobserver variability. With the XL probe, the determination of fibrosis can be enhanced in obese patients as well (48).

Recently, magnetic resonance elastography has come under the spotlight, since it seems to be the most accurate non-invasive imaging method for evaluating liver fibrosis (49).

A radiation force-based imaging method—ARFI—is made possible with conventional B-mode ultrasonography, and it can be divided into point shear wave elastography and two-dimensional shear wave elastography (2D-SWE). The main advantages of these non-invasive screening techniques are that they are less operator-dependent and the failure rate is lower than in transient elastography (47).

Fibrosis indices, such as APRI, may be reliable markers in identifying severe liver fibrosis, but are not appropriate for recognizing the early stages of liver involvement (50). A combination of TE and APRI may be a useful and precise diagnostic tool for CFLD (11).

Fibrotest or FibroSure (BioPredictive, Paris, France) is a novel complex composite fibrosis index—a combination of five serum biochemical markers—which is simple to use. It has high applicability, interlaboratory reproducibility, and comprehensive availability. However, its accuracy in detecting an intermediate level of fibrosis is limited, and it is not widespread in the clinical practice. In addition, it is expensive and has less specificity for liver disease (51).

Other fibrosis markers, Forns and AAR, are composite indices with the advantage of detecting advanced fibrosis and cirrhosis but not the early changes in liver structure (52).

An increasing number of studies have reported the pivotal role of TIMP-4 and endoglin in liver fibrosis. They could therefore aid in identifying liver involvement, although further studies need to be conducted to confirm their feasibility.

To summarize, our results indicate that the New criteria proposed by Koh et al. represent the relatively best diagnostic algorithm for detecting CFLD based on superiority indices and that transient elastography alone or in combination with TIMP-4 is ranked as the best screening technique. Furthermore, the New criteria show the highest pooled sensitivity in detecting CFLD (94%, [95% CI: 58–100]). Our analysis confirms that combined tests prove to have higher sensitivity but lower specificity, while single tests show higher specificity but lower sensitivity. The use of combined tests can increase the sensitivity of the testing, so this strategy can identify more patients with CFLD.

### Strength of the Study

A new statistical method was used for the diagnostic network meta-analysis, which provides a holistic evaluation of the index tests in the detection of CFLD. To our knowledge, this is the largest cohort of CF patients in an evaluation of the diagnostic accuracy of non-invasive techniques for detecting CFLD. Nonetheless, the strength of our meta-analysis is the homogeneous selection of the study population and the use of a comprehensive and precise search strategy and data extraction procedure.

### Limitations

Limitations include the minor differences between each of the domains of the composite reference standard. Although physical examination, liver biochemistry and radiological testing are all part of the Debray, the EuroCare and the Colombo diagnostic criteria, but they vary in the characterisations of the subsections. Furthermore, liver biopsy might be indicated in the Debray criteria if there is a doubt about a CFLD diagnosis, liver biopsy is not part of the diagnostic process in the other criteria.

Divergent use of cut-off values in the same index tests might increase the overrepresentation of the subjects; therefore, to conduct the DTA-NMA, we needed to design a transparent algorithm to choose the best cut-off values for the non-invasive tests. Moreover, cut-off values might vary according to age, gender, testing device etc. The index tests were also not used uniformly across the studies.

The populations of the studies were heterogeneous as regards mean age, since we included records with pediatric and adult populations. A further limitation is the inclusion of abstracts, a retrospective study and a case-control study in the analysis, thus possibly reducing quality evidence, and the risk of bias was unclear in the majority of the records.

However, there have been links between the development or severity of CFLD and specific *CFTR* mutations (the F508del homozygous *CFTR* genotype) and modifier genes in CFLD (e.g., the *SERPINA1 Z* allele) (49); there were data on genetics in only 40% of the studies.

In addition, due to the low number of participants in the studies, we cannot diminish the margin of statistical error, thus reducing the predictive power of our study, as indicated by the wide confidence intervals.

Potentially overlapping populations (33, 34) might distort the statistical analysis.

### Conclusions

#### Implications for Practice

The New criteria had the best diagnostic performance as well as the highest sensitivity in detecting CFLD. The second best option was transient elastography in an absolute competition between the tests: it preceded all the other imaging methods examined. Among the biochemical markers/fibrosis indices, the Forns index was ranked the highest. TE was more specific to CFLD, and the New criteria were more sensitive. These results raise the question whether the New criteria can serve as a proxy for the current gold standard. Further, TE, an easy-to-use and widely accessible modality, seems to outperform the more expensive state-of-the-art diagnostic modalities, so including TE in the current guidelines may be considered. An early diagnosis of CFLD allows early treatment initiation, which can prolong life expectancy.

#### Implications for Research

Due to the limitations of the evidence, our findings should be confirmed by future diagnostic accuracy studies. DTA-NMAs do not allow an index test to be better than the reference standard, so that other methods are called for to test if the New criteria can replace the current standard. Further, other non-invasive diagnostic modalities are worth investigating.

## Data Availability Statement

The original contributions presented in the study are included in the article/Supplementary Material, further inquiries can be directed to the corresponding author/s.

## Author Contributions

ZS and AP: study concept and design. ÁM, VI, and PP: acquisition of data. ÁM, VI, PP, and MI: analysis and interpretation of data. ÁM and ZS: drafting of the manuscript. ZS, ZR, and SV: critical revision of the manuscript for important intellectual content. AS: statistical analysis. AP and PH: study supervision. AP: the guarantor of this study, had full access to all the data and takes responsibility for the integrity of the data and the accuracy of the data analysis. All authors contributed to the article and approved the submitted version. Prior presentation information: We hereby state that the article has not been published and is not under consideration for publication elsewhere.

## Conflict of Interest

The authors declare that the research was conducted in the absence of any commercial or financial relationships that could be construed as a potential conflict of interest.

## Publisher's Note

All claims expressed in this article are solely those of the authors and do not necessarily represent those of their affiliated organizations, or those of the publisher, the editors and the reviewers. Any product that may be evaluated in this article, or claim that may be made by its manufacturer, is not guaranteed or endorsed by the publisher.

## Abbreviations

2D-SWE: two-dimensional shear wave elastography
AAR: AST-to-ALT ratio
ALP: alkaline phosphatase
ALT: alanine aminotransferase
APRI: AST-to-platelet-ratio index
ARFI: acoustic radiation force impulse
AST: aspartate aminotransferase
cAMP: cyclic adenosine monophosphate
CF: cystic fibrosis
CFLD: cystic fibrosis-related liver disease
CFTR: cystic fibrosis transmembrane conductance regulator protein
DOR: diagnostic odds ratio
DTA: diagnostic test accuracy
DTA-NMA: diagnostic test accuracy network meta-analysis
FIB-4: fibrosis-4 index
GGT: γ glutamyl-transferase
SI: superiority index
TE: transient elastography
TIMP-4: tissue inhibitor of metalloproteinase-4
US: ultrasonography.
