# Supplementary material for: Non-invasive Diagnostic Tests in Cystic Fibrosis-Related Liver Disease: A Diagnostic Test Accuracy Network Meta-Analysis
Source: Front Med (Lausanne). 2021 Jul 27;8:598382. doi: 10.3389/fmed.2021.598382 (PMC8353091; doi:10.3389/fmed.2021.598382)
Supplement: Supplementary file 1 [file Data_Sheet_1.ZIP › Suppl. TABLE 3.docx]

| **Diagnostic criteria** | **the EuroCare [1, 2]** | **the Colombo [3]** | **the Debray [1]** | **the New Criteria [4]** |
| --- | --- | --- | --- | --- |
| Diagnosis of CFLD should be considered if **one** of the following is present: | | | | |
|  |  |  |  | - Liver biopsy demonstrating pathology - Radiologic evidence demonstrating diffuse liver disease or cirrhosis. |
| Diagnosis of CFLD should be taken into consideration if **≥2** categories are present: | | | | |
| **Physical examination** | - Hepatomegaly and/or - Splenomegaly | Clinical hepatomegaly (increase in liver span and consistency, with liver edge palpable more than 2 cm below the costal margin on the mid-clavicular line), confirmed by ultrasonography. | - Hepatomegaly is defined as an increased liver span relative to age or as a liver edge palpable more than 2 cm below the costal margin on the mid-clavicular line, confirmed by ultrasonography. A prominent left lobe palpable in the epigastrium is often noted in cases of multilobular cirrhosis.   And/or   - Splenomegaly, confirmed by US. | Not part of the diagnostic criteria. |
| **Liver biochemistry** | Persistent abnormal liver biochemistry over 12 months. | Abnormal serum liver enzyme levels, consisting of elevation above the upper normal limits of 2 of the following: (AST, ALT and GGT). | Abnormalities of liver function tests defined as an increase of transaminases (AST and ALT) and GGT levels above the upper normal limits at least at 3 consecutive determinations over 12 months after excluding other causes of liver disease. | At least 2 persistently abnormal (*which is defined as having abnormal values on multiple dates over at least 2 consecutive years) ALT, AST, GGT or ALP. |
| **Radiological testing** | Ultrasound abnormalities. | Ultrasound abnormalities other than hepatomegaly (i.e., increased, heterogeneous echogenicity, nodularity, irregular margins, splenomegaly). Ultrasonographic pattern of steatosis did not represent a diagnostic criterion. | Ultrasonographic evidence of liver involvement (increased and/or heterogeneous echogenicity, irregular margins, nodularity) or portal hypertension (splenomegaly, in- creased thickness of the lesser omentum, spontaneous splenorenal anastomosis, large collateral veins, ascites) or biliary abnormalities (bile duct dilatation). | Evidence of hepatomegaly, splenomegaly, or portal hypertension by imaging. |
| **Liver biopsy** | Not part of the diagnostic criteria. | Not part of the diagnostic criteria. | A liver biopsy may be indicated if there is diagnostic doubt. | Not part of the diagnostic criteria. |
| **Non-invasive ultrasound-based techniques** | Not part of the diagnostic criteria. | Not part of the diagnostic criteria. | Not part of the diagnostic criteria. | Abnormal Fibroscan at any time. |
| **Non-invasive markers for fibrosis** | Not part of the diagnostic criteria. | Not part of the diagnostic criteria. | Not part of the diagnostic criteria. | Persistently abnormal* APRI, FIB-4, or AAR. |

**Supplementary Table 3: Diagnostic criteria for CFLD by „ the EuroCare”, „ the Colombo”, „ the Debray” and „the New criteria”.**

**Abbreviations:** US: Ultrasonography, AST: Aspartate aminotransferase, ALT: Alanine aminotransferase, GGT: γ glutamyl-transferase, ALP: Alkaline phosphatase, APRI: AST-to-platelet-ratio index, FIB-4: Fibrosis-4 index, AAR: AST-to-ALT ratio

**References**

1. Debray D, Kelly D, Houwen R, Strandvik B, Colombo C: **Best practice guidance for the diagnosis and management of cystic fibrosis-associated liver disease.** *J Cyst Fibros* 2011, **10 Suppl 2:**S29-36.

2. Lam S, Nettel-Aguirre A, Van Biervliet S, Roeb E, Sadler MD, Friedrich-Rust M, Karlas T, Kitson MT, deBruyn JCC: **Transient Elastography in the Evaluation of Cystic Fibrosis-Associated Liver Disease: Systematic Review and Meta-analysis.** *J Can Assoc Gastroenterol* 2019, **2:**71-80.

3. Colombo C, Battezzati PM, Crosignani A, Morabito A, Costantini D, Padoan R, Giunta A: **Liver disease in cystic fibrosis: A prospective study on incidence, risk factors, and outcome.** *Hepatology* 2002, **36:**1374-1382.

4. Koh C, Sakiani S, Surana P, Zhao X, Eccleston J, Kleiner DE, Herion D, Liang TJ, Hoofnagle JH, Chernick M, Heller T: **Adult-onset cystic fibrosis liver disease: Diagnosis and characterization of an underappreciated entity.** *Hepatology* 2017, **66:**591-601.
