# Supplementary material for: Non-invasive Diagnostic Tests in Cystic Fibrosis-Related Liver Disease: A Diagnostic Test Accuracy Network Meta-Analysis
Source: Front Med (Lausanne). 2021 Jul 27;8:598382. doi: 10.3389/fmed.2021.598382 (PMC8353091; doi:10.3389/fmed.2021.598382)
Supplement: Supplementary file 1 [file Data_Sheet_1.ZIP › Figure1/Suppl. Figure 1 legend.docx]

**Supplementary Figure 1. Risk of bias and applicability assessment**

**Abbreviations:** TE: Transient elastography, APRI: AST-to-platelet-ratio index, AAR: AST-to-ALT ratio, 2D-SWE: Two-dimensional shear wave elastography, US: Ultrasonography, ARFI: Acoustic radiation force impulse, TIMP-4: Tissue inhibitor of Metalloproteinase-4, ALP: Alkaline phosphatase.
