# Supplementary material for: Non-invasive Diagnostic Tests in Cystic Fibrosis-Related Liver Disease: A Diagnostic Test Accuracy Network Meta-Analysis
Source: Front Med (Lausanne). 2021 Jul 27;8:598382. doi: 10.3389/fmed.2021.598382 (PMC8353091; doi:10.3389/fmed.2021.598382)
Supplement: Supplementary file 1 [file Data_Sheet_1.ZIP › Suppl. TABLE 2.docx]

| **MEDLINE** | |
| --- | --- |
| #**1.** | "cystic fibrosis"[MeSH Terms] |
| #**2.** | cystic fibrosis [Text Word] |
| #**3.** | mucoviscidosis |
| #**4.** | liver |
| #**5.** | (#1 OR #2 OR #3) AND #4 |
| ("cystic fibrosis"[MeSH Terms] OR cystic fibrosis [Text Word] OR mucoviscidosis) AND liver | |
| Number of Results: 2261 | Search Date: October 8, 2019 |
| **EMBASE** | |
| #**1.** | ‘cystic fibrosis’ |
| #**2.** | mucoviscidosis |
| #**3.** | liver |
| #**4.** | (#1 OR #2 ) AND #3 |
| ('cystic fibrosis':ti,ab,kw OR mucoviscidosis:ti,ab,kw) AND liver:ti,ab,kw | |
| Number of Results: 2515 | Search Date: October 8, 2019 |
| **CENTRAL** | |
| #**1.** | cystic fibrosis |
| #**2.** | mucoviscidosis |
| #**3.** | liver |
| #**4.** | (#1 OR #2) AND #3 |
| ('cystic fibrosis':ti,ab,kw OR mucoviscidosis:ti,ab,kw) AND liver:ti,ab,kw | |
| Number of Results: 279 | Search Date: October 8, 2019 |
| **WEB OF SCIENCE** | |
| #**1.** | cystic fibrosis |
| #**2.** | mucoviscidosis |
| #**3.** | liver |
| #**4.** | (#1 OR #2) AND #3 |
| ('cystic fibrosis':ti,ab,kw OR mucoviscidosis:ti,ab,kw) AND liver:ti,ab,kw | |
| Number of Results: 2574 | Search Date: October 8, 2019 |
| **SCOPUS** | |
| #**1.** | cystic fibrosis |
| #**2.** | mucoviscidosis |
| #**3.** | liver |
| #**4.** | (#1 OR #2) AND #3 |
| TITLE-ABS-KEY ( ( cystic AND fibrosis OR mucoviscidosis ) AND liver ) | |
| Number of Results: 4092 | Search Date: October 8, 2019 |

**Supplementary Table 2: Search strategy for cystic fibrosis-related liver disease.** Systematic search was conducted in five databases on 8 October 2019 with the search keys seen above.
