# Supplementary material for: Non-invasive Diagnostic Tests in Cystic Fibrosis-Related Liver Disease: A Diagnostic Test Accuracy Network Meta-Analysis
Source: Front Med (Lausanne). 2021 Jul 27;8:598382. doi: 10.3389/fmed.2021.598382 (PMC8353091; doi:10.3389/fmed.2021.598382)
Supplement: Supplementary file 1 [file Data_Sheet_1.ZIP › Suppl. TABLE 5.docx]

| **Ranking of the index tests** | **Index tests** | **SI**  **(95% CI)** | **Pooled Se %**  **(95% CI)** | **Pooled Sp %**  **(95% CI)** | **DOR (95% CI)** |
| --- | --- | --- | --- | --- | --- |
| **NETWORK A** | | | | | |
| **#1** | **the New criteria** | 16.22 (0.64–31) | 94 (58–100) | 72 (52–84) | 107911.03 (3.62–236746.13) |
| **#2** | **TE** | 10.66 (1.40–27) | 65 (56–74) | 88 (84–91) | 14.67 (8.22–23.79) |
| **#3** | **TE + TIMP-4** | 8.84 (0.03–35) | 78 (30–100) | 64 (18–95) | 40698.24 (0.32–48569.14) |
| **#4** | **ARFI right lobe** | 7.39 (0.22–25) | 54 (33–75) | 89 (65–96) | 14.01(2.21–42.40) |
| **#5** | **2D-SWE + APRI** | 6.45 (0.03–33) | 59 (17–94) | 72 (22–99) | 31.65(0.18–208.69) |
| **#6** | **TE + APRI** | 6.22 (0.03–31) | 72 (24–98) | 65 (22–95) | 26.90 (0.28–187.45) |
| **#7** | **TE + endoglin** | 5.32 (0.03–29) | 74 (28–98) | 60 (18–93) | 25.35 (0.28–154.53) |
| **#8** | **ARFI left lobe** | 4.28 (0.05–25) | 54 (24–80) | 81 (46–94) | 9.20 (0.72–33.29) |
| **#9** | **FORNS** | 4.21 (0.03–29) | 74 (25-99) | 55 (14–91) | 45.54 (0.16–309.80) |
| **#10** | **ALP** | 3.88 (0.03–27) | 59 (14–94) | 66 (19–96) | 11.42 (0.20–73.08) |
| **#11** | **TIMP-4** | 3.80 (7.47-29) | 56 (12-91) | 67 (23–96) | 10.01 (0.16–74.06) |
| **#12** | **Liver function tests**  **(AST, ALT and GGT)** | 3.59 (0.03–29) | 48 (10–89) | 72 (22–97) | 11.57 (0.12–86.09) |
| **#13** | **2D-SWE** | 3.28 (0.03–27) | 63 (17–95) | 61 (16–94) | 10.61 (0.18–76.87) |
| **#14** | **Fibrotest corrected by haptoglobin** | 3.03 (0.03-27) | 45 (10–85) | 72 (26–97) | 9.36 (0.14–52.09) |
| **#15** | **APRI** | 2.92 (0.11–19) | 56 (38–73) | 79 (55–92) | 6.81 (1.24–20.52) |
| **#16** | **FIBROTEST** | 2.52 (0.03–23) | 57 (22–87) | 68 (29–91) | 6.06 (0.36–29.63) |
| **#17** | **Endoglin** | 2.48 (0.03–23.05) | 60 (18–93) | 59 (17–92) | 7.78 (0.15–50.80) |
| **#18** | **TIMP-4 + endoglin** | 2.44 (0.03–23) | 70 (23–98) | 50 (11–88) | 13.75 (0.16–83.87) |
| **#19** | **US** | 2.20 (0.04–17) | 69 (37–90) | 63 (40–82) | 5.78 (0.77–21.14) |
| **#20** | **AAR** | 1.36 (0.03–15.05) | 50 (11–88) | 57 (15–93) | 4.36 (0.09–27.80) |
| **NETWORK B** | | | | | |
| **#1** | **the New criteria** | 4.29 (0.14-11) | 80 (54–94) | 78 (50–93) | 28.91 (2.51–108.80) |
| **#2** | **TE + TIMP-4** | 3.97 (0.11-11) | 81 (33–100) | 66 (19–96) | 89146.11 (0.46–68936.35) |
| **#3** | **2D-SWE + APRI** | 2.01 (0.09-9) | 61 (18–94) | 70 (23–99) | 31.57 (0.26–292.75) |
| **#4** | **TE + APRI** | 1.99 (0.09-9.05) | 69 (20–98) | 63 (17–94) | 24.04 (0.25–200.31) |
| **#5** | **TE + endoglin** | 1.73 (0.09-9) | 67 (17–98) | 60 (16–95) | 27.93 (0.12–160.67) |
| **#6** | **TIMP-4 + endoglin** | 1.05 (0.09-7) | 67 (22–98) | 53 (13–90) | 13.52 (0.18–92.13) |
| **NETWORK C** | | | | | |
| **#1** | **TE** | 3.85 (0.33-9) | 66 (57–72) | 88 (85–91) | 15.14 (3.55–22.98) |
| **#2** | **ARFI right lobe** | 2.33 (0.2-7) | 54 (33–74) | 88 (66–96) | 13.92 (1.97–42.76) |
| **#3** | **ARFI left lobe** | 1.60 (0.14-7) | 55 (23–81) | 82 (50–95) | 10.04 (0.81–37.91) |
| **#4** | **2D-SWE** | 1.34 (0.11-7) | 63 (16–94) | 61 (16–93) | 11.45 (0.15–90.90) |
| **#5** | **US** | 1.21 (0.11-5) | 69 (39–89) | 63 (33–83) | 5.64 (0.84–18.82) |
| **NETWORK D** | | | | | |
| **#1** | **FORNS** | 5.23 (0.08-17) | 72 (25–99) | 63 (16–94) | 49.7 (0.22–328.08) |
| **#2** | **APRI** | 4.36 (0.20-13) | 55 (41–68) | 83 (66–89) | 6.83 (2.05–14.34) |
| **#3** | **ALP** | 3.89 (0.08-15) | 63 (18–93) | 64 (19–95) | 12.52 (0.22–77.28) |
| **#4** | **TIMP-4** | 3.29 (0.07-15) | 59 (17–93) | 63 (18–96) | 10.55 (0.17–73.02) |
| **#5** | **Combination of liver function tests (ALT, AST and GGT)** | 2.84 (0.07-15) | 48 (10–88) | 71 (26–98) | 11.06 (0.15–79.99) |
| **#6** | **Endoglin** | 2.77 (0.07-13) | 62 (16–94) | 57 (16–94) | 7.75 (0.15–52.58) |
| **#7** | **Fibrotest** | 2.56 (0.08-13) | 60 (27–86) | 64 (34–90) | 4.96 (0.42–22.72) |
| **#8** | **Fibrotest corrected by haptoglobin** | 2.09 (0.07-13) | 48 (9–86) | 64 (22–97) | 7.63 (0.10–57.22) |
| **#9** | **AAR** | 1.16 (0.06-9) | 52 (9–87) | 47 (11–91) | 2.77 (0.04–16.02) |

**Supplementary Table 5. Ranking of the index tests.**

**Network A:** The first analysis includes all eligible studies (fifteen) with all the different index tests (fifteen) and the combination of them. The New criteria is relatively the best diagnostic modality in the detection of CFLD, while TE and the combination of TE + TIMP-4 proved to be the second and third best options. **Network B:** In the second network analysis, in which the New criteria and the combined index tests were evaluated, the New criteria took first place, while the combination of TE + TIMP-4 and 2D-SWE + APRI were ranked in second and third places. **Network C:** Network C shows the comparison of imaging based techniques. TE was ranked the highest followed by ARFI in the right and left lobes. **Network D:** This network represents the ranking of biochemical markers and fibrosis indices which revealed that Forns index seems to be relatively the best diagnostic method, while APRI and ALP took second and third places.

**Abbreviations:** SI: superiority index; Se: sensitivity; Sp: specificity; DOR: diagnostic odds ratio; TE: transient elastography; 2D-SWE: two dimensional shear wave elastography; APRI: aspartate aminotransferase-to-platelet ratio index; ARFI: acoustic radiation force impulse; ALP: alkaline phosphatase; AST: aspartate aminotransferase; ALT: alanine aminotransferase; GGT: γ-glutamyl transferase; US: ultrasonography; AAR: AST-to-ALT ratio.
