# Supplementary material for: Non-invasive Diagnostic Tests in Cystic Fibrosis-Related Liver Disease: A Diagnostic Test Accuracy Network Meta-Analysis
Source: Front Med (Lausanne). 2021 Jul 27;8:598382. doi: 10.3389/fmed.2021.598382 (PMC8353091; doi:10.3389/fmed.2021.598382)
Supplement: Supplementary file 1 [file Data_Sheet_1.ZIP › Suppl. TABLE 4.docx]

**Supplementary Table 4. Description of the different non-invasive tests**

**Table of contents:**

1. Imaging-based techniques
   1. Transient elastography (TE)
   2. Acoustic radiation force impulse (ARFI) in the right and left lobes
   3. Two-dimensional shear wave elastography (2D-SWE)
2. Indices for calculating non‐invasive fibrosis markers
   1. APRI
   2. AAR
   3. FIB-4
   4. FORNS index
   5. Fibrotest
   6. Fibrotest corrected by haptoglobin
   7. Hepascore
   8. TIMP-4
3. **Imaging-based techniques:**
4. ***Transient elastography (TE) - Fibroscan ® (Echosens, Paris) [1-4]***

TE is a device that assess quantitative one-dimensional (1D) image of tissue stiffness which uses both ultrasound (US) (5 MHz) and low-frequency (50 Hz) elastic waves, whose propagation velocity is directly related to elasticity.

Liver stiffness is evaluated by measuring the velocity of a vibration wave generated on the skin. Shear wave velocity is determined by measuring the time the vibration wave takes to travel to a particular depth inside the liver. The stiffer the tissue, the faster the shear wave is propagated.

To improve test reliability a minimum of 10 valid readings, with at least a 60% success rate and an interquartile range of ≤30% of the median value, are taken with the results expressed in kilopascals (kPa).

TE is painless, rapid (it takes less than 5 minutes) and easy to perform at the bedside or in outpatients clinic.

The main disadvantages of liver stiffness measurements are that it can be difficult to obtain in obese patients or in those who have narrow intercostal spaces, and is impossible to achieve in patients with ascites and it lacks B-mode grey scale ultrasound visualization of the liver.

Screening with TE is cost-effective with mean incremental cost-effectiveness ratios ranging from 2,570 €/QALY (95% CI 2,456–2,683) for a population at risk of alcohol-related liver disease (age ≥45 years) to 6,217 €/QALY (95% CI 5,832–6,601) in the general population.

1. ***Acoustic radiation force impulse (ARFI) in the right and left lobes [5, 6]***

It is a radiation force-based imaging method that is provided by conventional B-mode ultrasonography. ARFI imaging involves transmission of an initial ultrasonic pulse at diagnostic intensity levels to obtain a baseline signal for later comparison. A short-duration (approximately 0.3 s), high-intensity acoustic ‘pushing pulse’ is then transmitted by the same transducer, followed by a series of diagnostic intensity pulses, which are used to track the displacement of the tissue caused by the pushing pulse. Higher shear wave speeds and smaller displacements are associated with stiffer tissues, and slower shear wave speeds and larger displacements occur with more compliant tissues. The main advantages of the technique are that it does not require compression of the transducer, and is expected to be less operator dependent.

Mean costs for liver biopsy varied from £70,274 to £75,957 and mean effects varied from 9.64 QALYs to 11.41 QALYs. Among hepatitis B antigen-positive patients (high levels of the virus and greater infectiousness) costs for ARFI and TE were £83,487 and £79,004 and mean effects were 11.71 QALYs and 11.61 QALYs, respectively. Among hepatitis B antigen-negative patients (low to zero level of the virus and less infectious) costs for ARFI and TE were £77,512 and £73.007 and mean effects were 10.10 QALYs and 9.93 QALYs, respectively. ARFI was dominated by less costly and more effective options among hepatitis B antigen-positive patients; ARFI was extendedly dominated in hepatitis B antigen-negative patients.

1. ***Two-dimensional shear wave elastography (2D-SWE )[7, 8]***

ARFI techniques can be divided into point shear wave elastography (p-SWE) and two dimensional shear wave elastography (2D-SWE) techniques. 2D-SWE is an newer elastography technique which is integrated into ultrasound machines allowing a real-time visualization of liver structure and the ability to choose the region of interest, avoiding vessels and the liver capsule. It quantifies tissue stiffness by measuring the speed of shear waves induced into the tissue by acoustic push pulses, generating two-dimensional quantitative images of shear-wave speed. The advantages of this technique include real-time visualization of a colour quantitative elastogram superimposed on a B-mode image, enabling the operator to be guided by both anatomical and tissue stiffness information.

1. **Indices for calculating non‐invasive fibrosis markers:**
2. ***The AST‐to-platelet‐ratio‐index (APRI) [9]:***APRI = [AST (U/L) ÷ULN of AST (U/L)] ÷ [Platelet count (K/μl)]
3. ***The AST to ALT ratio (AAR) [10]:***

AAR = AST (U/L) ÷ ALT (U/L)

1. ***The Fibrosis‐4 index (FIB‐4) [11, 12]:***

FIB‐4 = Age (years) x AST (U/L) ÷ Platelet count [(10(9)/L] x [ALT (U/L)] 1/2

1. ***The FORNS index [13]:***

7.811-3.131 × ln Platelet count [10(9)/L)] + 0.781 × ln [GGT(IU/L)] + 3.467 × ln (age [years]) - 0.014 × ln [cholesterol (mg/dl)]

1. ***The FIBROTEST ® (FibroSure) [14] :***

Formula combining α2-macroglobulin, γ-GT, Apolipoprotein A1, Haptoglobin, Total bilirubin, Age and Gender.

The equation for calculating the FibroTest score regression coefficient is:

z= 4.467 x log_10_[α2-macroglobulin (g/L)] – 1.357 z=4.467×log10⁡[Alpha2M(g/L)]−1.357×log10⁡[Haptoglobulin(g/L)]+1.017×log10⁡[GGT(IU/L)]+0.0281×[Age]+1.737×log10⁡[BIL(μmol/L)]−1.184×[Apoliprotein(g/L)]+0.301×B−5.54x log_10_[Haptoglobin (g/L)] + 1.017 x log_10_ [GGT (IU/L)] + 0.0281 x [Age] + 1.737 x log_10_[Bilirubin (μmol/L)] – 1.184 [Apoprotein (g/L)] + 0.301 x B – 5.54

where B=1 for male and B=0 for female.

The score is between 0 and 1

F=1/(1+e−z)

1. ***The FIBROTEST corrected by haptoglobin [15]:***

The formula is corrected by using the median of haptoglobin (0.89 g/l).

1. ***The HEPASCORE[16]:***

*y* = exp [4.185818 – (0.0249 x age) + (0.7464 x sex) + (1.0039 x α 2-macroglobulin) + (0.0302 x hyaluronic acid) + (0.0691 x bilirubin) – (0.0012 x GGT)]

with age provided in years, male sex = 1, female sex = 0, α 2-macroglobulin in g/L, hyaluronate in μg/L, bilirubin in μmol/L, and GGT in U/L.

1. ***TIMP-4 [17]:***

Tissue inhibitor of metalloproteinase-4 (TIMP-4) belongs to a family of extracellular matrix (ECM) metalloproteinases inhibitors that are overexpressed in several cancers.

**Abbreviations:** TE: Transient elastography, US: Ultrasonography, KPA: Kilopascals, CI: Confidence interval, ARFI: Acoustic radiation force impulse, AST: Aspartate aminotransferase, ALT: Alanine aminotransferase, GGT: γ glutamyl-transferase, ALP: Alkaline phosphatase, APRI: AST-to-platelet-patio index, FIB-4: Fibrosis-4 index, AAR: AST-to-ALT ratio, TIMP-4: Tissue inhibitor of metalloproteinase-4, ECM: Extracellular matrix

**References**

1. Sandrin L, Fourquet B, Hasquenoph JM, Yon S, Fournier C, Mal F, Christidis C, Ziol M, Poulet B, Kazemi F, et al: **Transient elastography: a new noninvasive method for assessment of hepatic fibrosis.** *Ultrasound Med Biol* 2003, **29:**1705-1713.

2. Castera L, Forns X, Alberti A: **Non-invasive evaluation of liver fibrosis using transient elastography.** *J Hepatol* 2008, **48:**835-847.

3. de Lédinghen V VJ: **Transient elastography (FibroScan).** *Gastroenterol Clin Biol* 2008, **32(6 Suppl 1):58‐67**.

4. Serra-Burriel M, Graupera I, Torán P, Thiele M, Roulot D, Wai-Sun Wong V, Neil Guha I, Fabrellas N, Arslanow A, Expósito C, et al: **Transient elastography for screening of liver fibrosis: Cost-effectiveness analysis from six prospective cohorts in Europe and Asia.** *J Hepatol* 2019, **71:**1141-1151.

5. Nightingale K: **Acoustic Radiation Force Impulse (ARFI) Imaging: a Review.** *Curr Med Imaging Rev* 2011, **7:**328-339.

6. **CADTH Rapid Response Reports.** In *Acoustic Radiation Force Impulse Imaging for Diagnosis and Monitoring of Liver Fibrosis in Patients with Hepatitis C: A Review of Diagnostic Accuracy, Clinical Effectiveness, Cost-Effectiveness, and Guidelines.* Ottawa (ON): Canadian Agency for Drugs and Technologies in Health

Copyright © 2016 Canadian Agency for Drugs and Technologies in Health.; 2016.

7. Bende F, Sporea I, Sirli R, Popescu A, Mare R, Miutescu B, Lupusoru R, Moga T, Pienar C: **Performance of 2D-SWE.GE for predicting different stages of liver fibrosis, using Transient Elastography as the reference method.** *Med Ultrason* 2017, **19:**143-149.

8. Shiina T, Nightingale KR, Palmeri ML, Hall TJ, Bamber JC, Barr RG, Castera L, Choi BI, Chou YH, Cosgrove D, et al: **WFUMB guidelines and recommendations for clinical use of ultrasound elastography: Part 1: basic principles and terminology.** *Ultrasound Med Biol* 2015, **41:**1126-1147.

9. Wai CT, Greenson JK, Fontana RJ, Kalbfleisch JD, Marrero JA, Conjeevaram HS, Lok AS: **A simple noninvasive index can predict both significant fibrosis and cirrhosis in patients with chronic hepatitis C.** *Hepatology* 2003, **38:**518-526.

10. Sheth SG, Flamm SL, Gordon FD, Chopra S: **AST/ALT ratio predicts cirrhosis in patients with chronic hepatitis C virus infection.** *Am J Gastroenterol* 1998, **93:**44-48.

11. Sterling RK, Lissen E, Clumeck N, Sola R, Correa MC, Montaner J, M SS, Torriani FJ, Dieterich DT, Thomas DL, et al: **Development of a simple noninvasive index to predict significant fibrosis in patients with HIV/HCV coinfection.** *Hepatology* 2006, **43:**1317-1325.

12. Vallet-Pichard A, Mallet V, Nalpas B, Verkarre V, Nalpas A, Dhalluin-Venier V, Fontaine H, Pol S: **FIB-4: an inexpensive and accurate marker of fibrosis in HCV infection. comparison with liver biopsy and fibrotest.** *Hepatology* 2007, **46:**32-36.

13. Forns X, Ampurdanès S, Llovet JM, Aponte J, Quintó L, Martínez-Bauer E, Bruguera M, Sánchez-Tapias JM, Rodés J: **Identification of chronic hepatitis C patients without hepatic fibrosis by a simple predictive model.** *Hepatology* 2002, **36:**986-992.

14. Imbert-Bismut F, Ratziu V, Pieroni L, Charlotte F, Benhamou Y, Poynard T: **Biochemical markers of liver fibrosis in patients with hepatitis C virus infection: a prospective study.** *Lancet* 2001, **357:**1069-1075.

15. Friedrich-Rust M, Schlueter N, Smaczny C, Eickmeier O, Rosewich M, Feifel K, Herrmann E, Poynard T, Gleiber W, Lais C, et al: **Non-invasive measurement of liver and pancreas fibrosis in patients with cystic fibrosis.** *Journal of Cystic Fibrosis* 2013, **12:**431-439.

16. Adams LA, Bulsara M, Rossi E, DeBoer B, Speers D, George J, Kench J, Farrell G, McCaughan GW, Jeffrey GP: **Hepascore: an accurate validated predictor of liver fibrosis in chronic hepatitis C infection.** *Clin Chem* 2005, **51:**1867-1873.

17. Melendez-Zajgla J, Del Pozo L, Ceballos G, Maldonado V: **Tissue inhibitor of metalloproteinases-4. The road less traveled.** *Mol Cancer* 2008, **7:**85.
