# Supplementary figures and images for: Non-invasive Diagnostic Tests in Cystic Fibrosis-Related Liver Disease: A Diagnostic Test Accuracy Network Meta-Analysis
Source: Front Med (Lausanne). 2021 Jul 27;8:598382. doi: 10.3389/fmed.2021.598382 (PMC8353091; doi:10.3389/fmed.2021.598382)

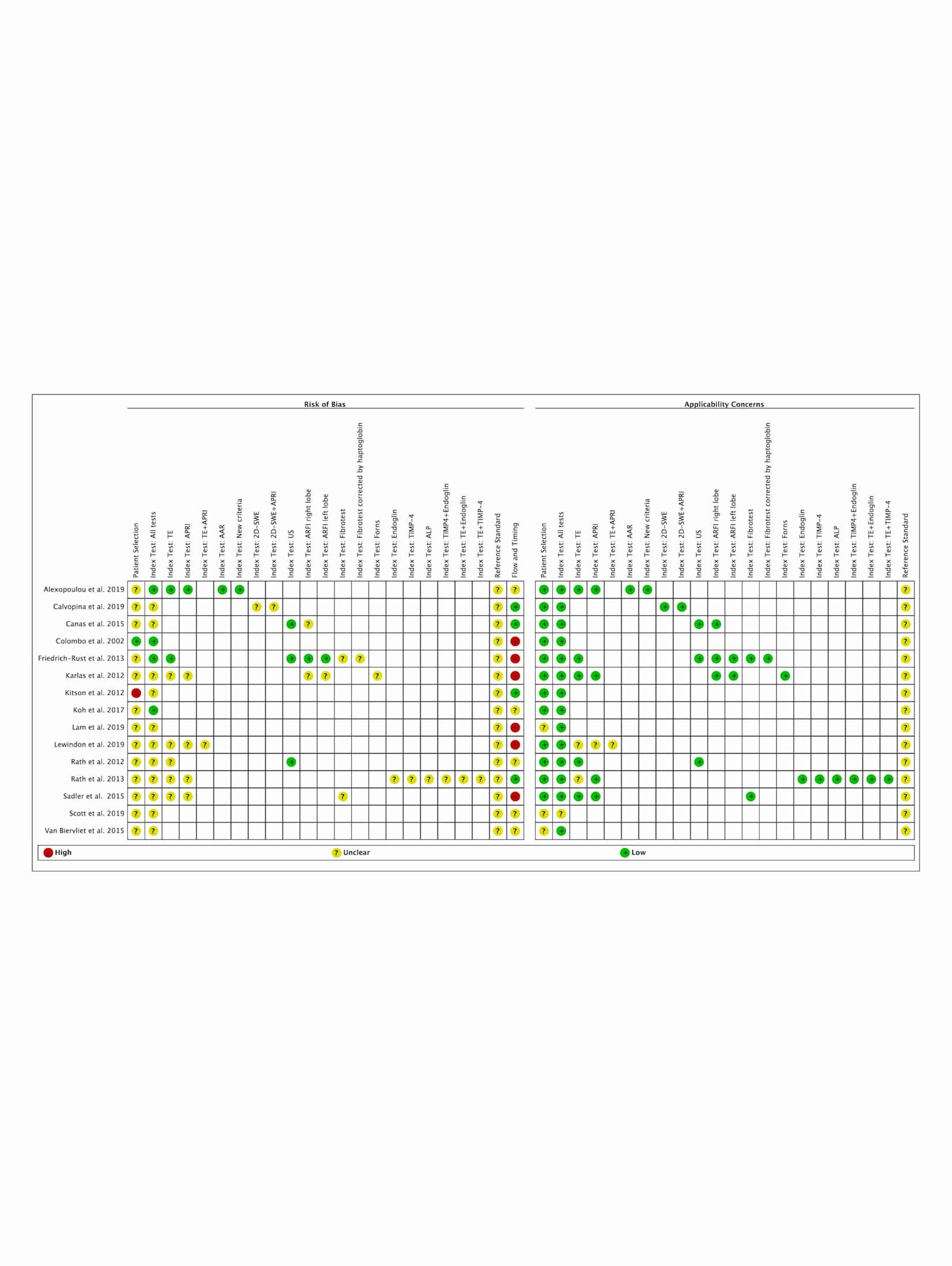

Supplement: Supplementary file 1 [file Data_Sheet_1.ZIP › Figure1/Suppl. FIGURE 1.jpg]

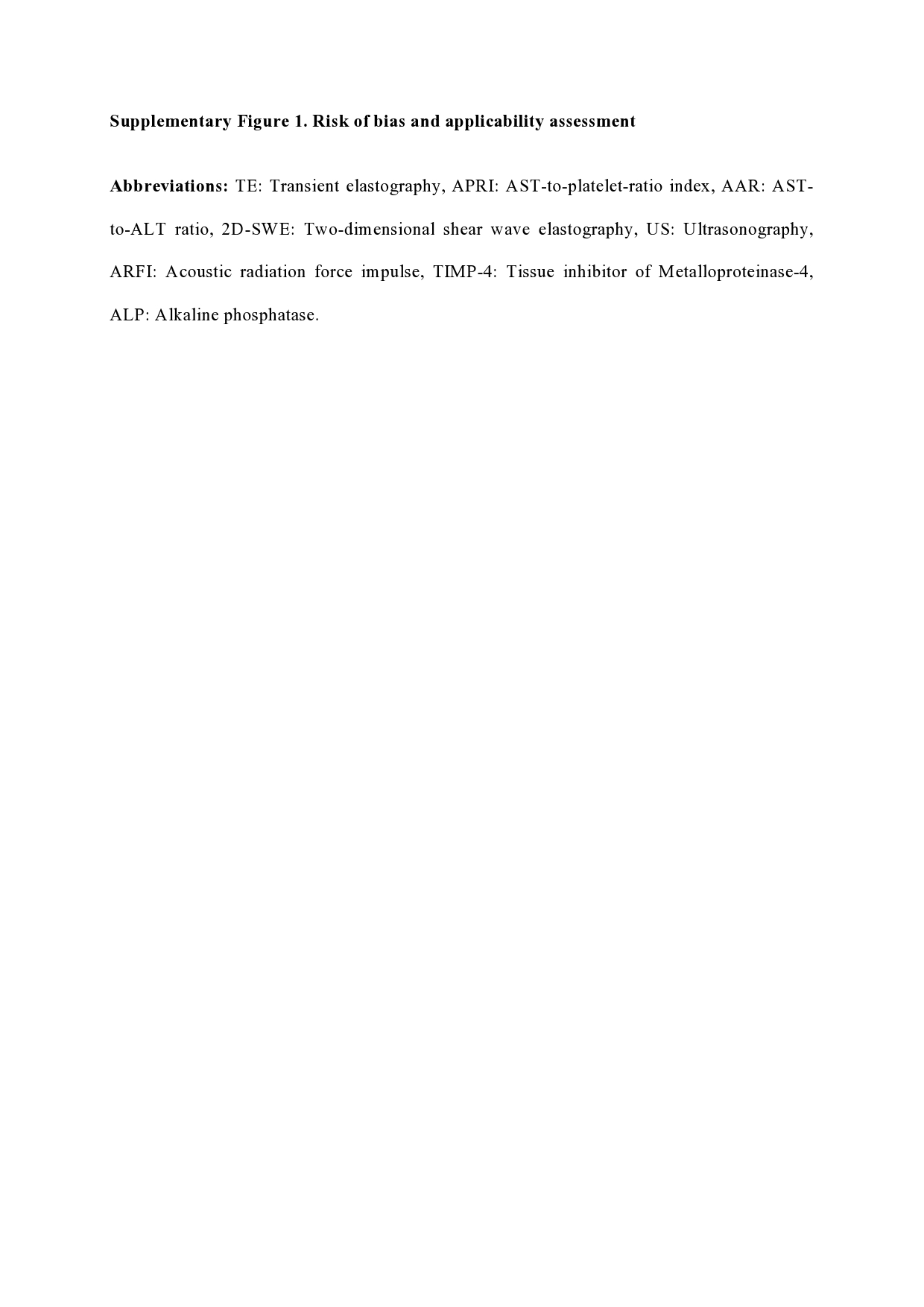

Supplement: Supplementary file 1 [file Data_Sheet_1.ZIP › Figure1/Suppl. Figure 1 legend-page0001.jpg]
